# Three *Strigeid* cercariae from *Littorina littorea* snail, Qarun Lake, Fayoum, Egypt

**DOI:** 10.14202/vetworld.2018.310-315

**Published:** 2018-03-14

**Authors:** Fayez A. Bakry, Marwa Th. Atwa, Marwa M. Attia

**Affiliations:** 1 Department of Medical Malacology Theodor Bilharz Research Institute, Giza, Egypt; 2 Department of Zoology, Faculty of Science, Fayoum University, Fayoum Governorate, Egypt; 3 Department of Parasitology, Faculty of Veterinary Medicine, Cairo University, Giza, Egypt

**Keywords:** *Littorina littorea*, Qarun Lake, *Strigeid Cercaria*

## Abstract

**Aim:**

The present study aims to focus on the role of common marine snails (*Littorina littorea)* as a vector for some trematode parasites.

**Materials and Methods:**

A total of 327 marine water *L. littorea* snails were collected during the summer of 2016 from a Qarun lake in the EL-Fayoum Governorate, Egypt. The snails were investigated for infection by trematode parthenitae through induction of cercarial shedding by exposure to light and crushing the snails. The species were stored in Search Laboratory of Zoology Department, Faculty of Science, Fayoum University.

**Results:**

Three species of *Strigei*d *littorin*a *cercari*a were identified from the infected snails. They are described here and they identified in relation to close-up morphological features and linked to its snail hosts. They give the following names: *Cercaria strigeid littorina* type 1, *C. strigeid littorina* type 2, and *C. strigeid littorina* type 3. The incidence of infection by these cercariae was 33%, 25.7%, and 2.4%, respectively.

**Conclusion:**

This study is clarifying the importance of this marine snail as intermediate hosts for new trematode species.

## Introduction

Lake Qarun is the third largest lake in Egypt and the second most famous one after Lake Nasser in the Southern part of Egypt. It lies 45 m below sea level and occupies the lowest, northern section of the Fayoum depression. It is a marine lake containing different types of fish, snails, and seabirds along the year. The study of animal parasites in the Lake Qarun region began with the report of Al-Bassel [1] and Abdel-Ghaffar *et al*. [2,3].

*Littorina littorea* possesses an operculum that is used to close off the shell aperture on disturbance, analogous to the hiding responses and withdrawal responses seen in other animals [4]. Parasitic infection can alter the host behavior in ways that might be adapted for the parasite, with parasite-induced behavioral changes apparent at the sample mean level [5]. Parasites might impact behavior at the level of animal personalities (Hammond-Tooke *et al*. [6] and Poulin [7]) as well as at the sample mean. However, only a few studies [6,8,9] have investigated this possibility empirically.

Several authors still interested in studying the disease and its vectors in and around the lake. According to the water movement, large numbers of snails usually present more superficial around the lake boundaries and even on the ground. These snails considered an edible food for most of seabirds that fly around the lake and the snails are rather ingested by birds accidentally with stones. Inspection of these snails proved that they contain variable trematode parthenita that usually infects these birds as well as fish [10-12].

Huxham *et al*. [10] and Galaktionov and Dobrovolskij [12] revealed that *L. littorea* snail is one of these abundant snail species available to be picked up by seabirds as food. It is a marine gastropod mollusk (Phylum: Mollusca, Class: Gastropoda, Order: Prosobranchia, Suborder: Neotaenioglossa, Family: Littorinidae, Genus: *Littorin*a, Species *L. littore*a). It has gills and an operculum. It is 10-12 mm in maximum width and about 16-38 mm in length, with broadly oval shell. The snail is mainly found on rocky shores in the higher and middle internal zone. It sometimes lives in small tide pools. It may also be found in muddy habitats such as estuaries and can reach depths of 180 ft.

The parasite fauna infecting *L. littorea* has been investigated previously by Seaman and Briffa [13] and Pechenik *et a1*. [14]. They were diagnosed up to five species of trematodes (*Cryptocotyle lingua*, *Cercaria parvicaudata, Renicola roscovita*, *Microphallus pygmaeus*, and *Microphallus similes*) in *L. littorea*. This snail is the first intermediate host (IMH) to the larval stages of *Cryptocotyle lingua* and *C. parvicaudata*. The snails ingest eggs of both parasites deposited in the feces of gulls (*Larus* spp.). Cercariae are released from infected snails and infect the second IMH, as metacercariae encyst on fish skin while *C*. *parvicaudata* infects bivalve mollusk [14,15].

Role of snails as IMH for new parasites still needs more investigations aiming to determine the epidemiology of new parasites and determine the best way of controlling them [16,17].

With respect to the previous experiences of the authors in the field of trematode parthenitae in this locality, The aim of the current study was to spotlight on the role of the common marine snails (*L. littorea*) as a vector for some trematode parasites that can be transmitted from them to other hosts.

This research focused on the prevalence of cercariae in the snail. We recommended that further researches to be done about biological manipulation of the parasites as no studies about this issue in this locality reported previously and so little information is available about this.

We focused in this article on new species, but we found other species and we cannot find schistosomiasis during the period of the study.

## Materials and Methods

### Ethical approval

The Institutional Animal Ethics Committee and local laws and regulations were considered in applying our experiment.

### Study area

The present study was done on Qarun marine water lake at the North West region of El-Fayoum Governorate. The Governorate is an oasis in the western desert 100 km southwest of Cairo. It lies between Latitude of 29° 4’17” N and Longitude of 30° 7’12” E.

### Collected samples

*L. littorea* snails were collected manually according to the method described by Mandahi-Barth [18] using suitable long-handled net. The snails were separated from the associated submerged vegetation by several agitations in clean lake water. Collected snails were picked up manually and maintained in the laboratory during investigation in the same water in clean plastic aquaria supplied by its some vegetation. The snails were identified according to McQuaid [19].

### Inspection of snails

All collected snails were examined for trematode infection by light exposure and crushing techniques [20] as the following: The snails were investigated for the presence of infection by the exposure technique as 3-5 selected snails (10 replicates) which were placed in a Petri dishes half filled with clear filtered natural lake water. They exposed to a direct light using 100 watts electrical lambs for 1-2 h under observation. The normally shed cercariae were picked up directly and fixed for identification as well as for measurements. An alternative technique was used by crushing representative samples from the collected snails directly in suitable Petri dishes with a few amount of water under a dissecting microscope, where available parthenitae were recorded by El-Bahy *et al*. [20].

### Examination of the obtained stages

Fresh obtained living *Cercaria* and sporocysts were stained by supravital stain using dilute solutions vital dyes as Neutral red and Nile blue sulfate according to Cable [21]. Moreover, the dead relaxed specimens were fixed and stained for the preparation of permanent mounted using the technique described by Prichard and Kruse [22]. Detected trematode parthenita was identified according to Diab [23], then recorded for each snail species separately.

### Detailed inspection and measuring of the stages

During observation of shed cercariae, all biological characters were recorded including time of shedding, movement, and behavior of the cercariae from time of shedding till death or encystment. This was done on samples of directly shed cercariae after they transferred to a suitable Petri dish in enough amount of water under laboratory conditions (20-25°C).

For measuring the cercariae, they were fixed according to Cort and Brackett [24], where a small quantity of water containing the cercariae was placed in a container and then an equal volume of 10% formalin heated to boiling was added. A number of 25 well-relaxed moist-heat killed stages were measured.

All measurements were taken with the aid of eyepiece micrometer. Measurements were given in microns and both minimum and maximum figures were recorded in the description, with the average being given in parentheses.

Sporocysts were studied alive under the microscope. Longevity and other living habits of these larvae were also studied. Measurements were made from specimens fixed in 5% formalin and stained with ­acetic acid alum carmine.

## Results

**Figure-1:**
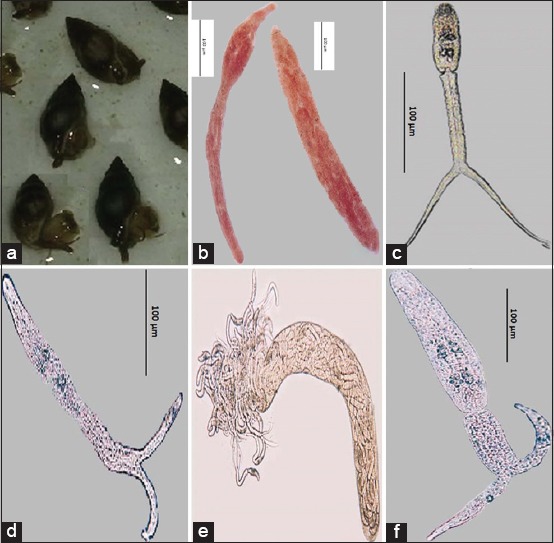
(a) *Littorina littorea* active snail, (b) Sporocyst contain *Cercaria strigeid littorina* type 1, (c) *C. strigeid littorina* type 1, (d) *C. strigeid littorina* type 2, (e) *Sporocyst contain C. strigeid littorina* type 2, (f) *C. strigeid littorina* type 3.

During the summer season of 2016, a number of 327 *L. littorea* snails (Figure-1a) were collected and inspected for diagnosing the rate of infection by different trematode parthenitae. As described in Table-1, three cercariae and their parthenitae were diagnosed in the inspected snail. They are characterized as follows:

**Table-1:** Distribution of the target Cercariae in the examined *L. littorea* snail.

*Cercaria* type	No. examined	No. infected	% and conditions of infection
*Cercaria strigeid*	327	109	33% - mixed inf.
*Littorina* type 1			
*Cercaria strigeid*	327	84	25.7% - mixed
*Littorina* type 2			
*Cercaria strigeid*	327	8	25.7% - mixed
*Littorina* type 3			

%=The percentage of infection (The number of infected snails related to the total number of examined snails)

### Conditions of infection

State either a snail was infected with mixed infection by more than one type of *Cercaria* (as type 1 and type 2 which were found together in the same snail) or infected with a single infection with only one type of *Cercaria* (as type 3 which was found singular in the snail).

### 1^st^ identified *Cercaria* and its sporocyst

#### Cercaria strigeid littorina type 1 (Figure-1b and c)

This *Cercaria* is a type of *Strigeid* cercariae, and it was diagnosed in 33% of the inspected snails. It is characterized by the body of the same width but considerably shorter than tail stem; furca is almost as long as the tail stem; The body measured 151-200 μ in length (mean of 175.5 μ) and 50-77 μ (mean 63.5 μ) in width. The distance from middle of ventral sucker to the posterior end of body was about 55-76 μ (mean 65.5 μ). The tail stem was about 290-330 μ (mean is 310 μ) length. Furca is long and was about 300-354 μ (mean 327 μ) in length.

The oral and ventral suckers were prominent and were approximately of the same diameter that measured 28-36 μ (mean=32 μ) length and about 27-34 μ (mean of 30.5 μ) width. The prepharynx was extremely short. The pharynx was short. Esophagus was moderately long and divided into two simple intestinal ceca extending to the level of posterior margin of the ventral sucker. One row of small spines encircles ventral sucker. Penetration glands, coarsely granular, eight in number, four are lying in front of and four are behind ventral sucker. Their ducts ran in sinuous course and opened at the tip of the body around the oral sucker.

Cercaria advanced inside Sporosest elongated (Figure-1c). The young sporocysts were narrow and tubular with a size of 0.225-0.391 mm (0.308 mm) and the length of 0.135-0.145 mm (0.14 mm) width.

Some sporocysts were as large as 3.11 mm length. In some cases, the width of the sporocyst was very uniform along its entire length (Figure-1b), but others had swollen portions connected by narrower regions (Figure-1b). The ends of sporocysts may be pointed or rounded, and no direct correlation was found between the size of sporocyst and the presence of *Cercaria* within the sporocyst.

When cercariae hang freely in water, tail stem was bent sharply to side and there was a marked constriction in posterior body.

Following up the previous description of similar cercariae, this type could be identified as a species from *Strigeid* cercariae, and concerning its snail IMH, the author suggests identifying this species as *C. strigeid littorina* type 1 (Figure-1b and c).

### The 2^nd^ identified *Cercaria* and its sporocyst

#### C. strigeid littorina type 2 (Figure-1d and e)

It was another type of large *Strigeid Cercaria*. It infected 25.7% of the examined snails. It was usually present in mixed infection with the first type. Its body was 255-290 μ (272.5 μ) length and 44-52 μ (48.0 μ) width. Tail stem was slightly shorter than body length, which measured 205-275 μ (mean of 240 μ) length and 41-51 μ (mean 46 μ) width.

The length of the furcal was 189-220 μ (204.5 μ). The oral sucker was wide in living specimens, elongation when fixed and slightly larger than a ventral sucker, measuring 44-51 μ (47.5 μ) in the and 25-28 μ (26.5 μ) in width, while ventral sucker measured 32-37 μ (34.5 μ) in length and 18-23 μ (20.5 μ) in width. Pharynx was small, esophagus was narrow, and intestinal cake was extended to ventral sucker. Penetration glands were small, very inconspicuous and eight in number, four are lying in front of ventral sucker and the others are behind the ventral sucker. Gland ducts ran forward and opened around the oral sucker.

The body surface was armed with spines, and most of them had 12 transverse rows which had a weakly scalloped appearance to the body surface during body contraction. Ventral sucker had two to three rows of spines.

The tail stem surface, particularly, the anterior surface was covered with small spines. The digestive system was well developed. The prepharynx was short leads to the short pharynx, while the length of the esophagus was 6-8 times the length of the pharynx. The esophagus ended at a bifurcation located a short distance anterior to the ventral sucker, and from this point, the ceca extended laterally and posteriorly to the excretory bladder.

The examination on living material showed that a birth pore, through which the cercariae emerged, was present at one end of the sporocyst (Figure-1e).

Swimming activity was not affected by exposure to different light intensities. Swimming activity was in the form of very rapid whipping of tail back and forth followed by a period of inactivity during which the *Cercaria* was suspended, body downward, in the water.

Following up the previous description of similar cercariae, this type could be identified as a species from *Strigeid Cercaria* and concerning its snail IMH. The author suggests identifying this species as *C. strigeid littorina* type 2 (Figure-1c).

### The 3^rd^ identified *Cercaria*

#### C. strigeid littorina type 3 (Figure-1f)

It is another type of *Strigeid Cercaria*. It was diagnosed in just 2.4% of the inspected snail. It was present in single infection not associated with other types. Its body was 230-265 μ length (mean, 247.5 μ) and 55-67 μ width (mean 61.0 μ).

Tail stem was clearly shorter than body length measured 120-134 μ length (mean, 127.0 μ) and 45-51 μ width (mean, 48.0 μ), furca elongate, contractile measured 118-130 μ (mean, 124.0 μ). Oral sucker was elongate and slightly larger than ventral sucker measured 48-53 μ (mean, 50.0 μ) length and 31-37 μ (mean, 34.0 μ) width, while ventral sucker measured 41-47 μ (44.0 μ) length and 25-32 μ (mean, 28.5 μ) width. Pharynx was slightly larger, and esophagus was short and leads to two intestinal ceca. Penetration glands were small and twelve in number, six lying in front of and six are behind the ventral sucker. Ventral sucker had several irregular rows of flattened spines of various sizes. Excretory system was typical of strigeids. Three flame cells were on each anterior collecting tubule. Five flame cells were found on each posterior collecting tubule. No caudal bodies were present in tail stem.

The emergence and activity of this species were not studied adequately because the cercariae emerged in small number. The cercariae emerged during most of the day, but shedding occurred mainly in the afternoon. After resting for varying periods of time, they swam upward and vertically. When they stopped, the furca was held at right angles to the tail stem. Then, the furca gradually assumed a position at right angles to each other (Figure-1f).

Following up the previous description of similar cercariae, this type could be identified as a species from *Strigeid Cercaria*, and concerning its snail IMH, the authors suggest identifying this species as *C. strigeid littorina* type 3 (Figure-1f).

## Discussion

The snail *L. littorea* is a marine water snail, inhabits the Lake Qarun, and usually lives above the mean high tide line. The snails are occasionally present in very large numbers as they are easy to be collected. Collection and examination of 327 *Littorea* sp., 10-14 mm length during the summer of 2016, revealed infection by sporocysts and cercariae in 33% of the snails with *C. streiged littorina* type 1.

This *Cercaria* has an excretory system with clear commissure that developed and located at the posterior end of the body. Excretory duct runs in the middle of tail stem and along the furca to open on their tips. Tail stem has a limited number of large, uniform caudal bodies.

The recorded *C. strigeid littorina* type 1 in the present study was characterized by having a limited number of large, rather uniform caudal bodies in the tail stem as in *Cercaria emarginatae* Cort [25] and *Cercaria dohema* Cort and Brackett [24], but this *Cercaria* is distinguished from them by its characteristic form and number of its penetration gland and also unusually long furcae.

From numerous previously described species of *Strigeid* cercariae, two species, *Cercaria macradena* and *C*. *microdena* Cort and Bracket [26], obtained from Douglas Lake region from *Stagnicola palustris* snails, are similar morphologically to our *Cercaria* that identified as *C. strigeid littorina* type 2 as it having penetration gland in front and behind the ventral sucker, excretory pattern, and body spinabion, but differ significantly in size, proportions, and the number and size of penetration glands.

The obtained Strigeid littorina cercaria type 2 in the present study was similar to cercaria higginsi [27] which obtained from lymnaeid snails collected in northern Michigan in morphology, size, proportions, where the body and tail stem are very approximately equal in size, and the hanging in the water but differ significantly in having eight penetration glands in front and behind the ventral sucker. While *C. higginsi* has six small penetration gland posterior to ventral sucker and also the tail stem has no caudal bodies.

A single snail was infected with both *C*. *strigeid littorina* type 1 and *C. strigeid littorina* type 2. This result does not provide evidence of competitive trematode interactions within *L. littoria* [28], but the typical cooccurrence of two parasites in any snail would be rare [29]. The 3^rd^ species that give the name of *C. strigeid littorina* type *3* has all characteristic features of the cercariae belonging to the family Strigeidae. This *Cercaria* is similar to *Cercaria sincera* which described by Olivier [27] in the dimensions of tail stem and furca and in having the same structure of the excretory system. However, *C. strigeid littorina* type 3 was characterized chiefly by its penetration glands, it has six lying in front and six behind the ventral sucker, while *C. sincera* has two pairs of small penetration glands lateral or anterior to the ventral sucker. The present *Cercaria* distinguished also by its unusual short tail stem if compared with body length where it is smaller 1½ times the length of the body.

Among 327 specimens of *L. littorea* snails collected on May 2016 from Lake Qarun, eight snails only (2.4%) were infected with this species of *Cercaria* where the *Cercaria* was found in single infection, unlike the two previous species (*C. strigeid littorina* type 1 and *C. strigeid littorina* type 2) where they were found in double infect. The predominance of infections by one parasite (*C. strigeid littorina* type 3) could be suggest that *C*. *strigeid littorina* type 3 determines the outcome of interactions by arriving first and determine other infections [30] or that *C*. *strigeid littorina* type 3 is the competitive dominant regardless of which parasite infects the host first [28]. Possibilities also include immunological responses or cellular tissue reactions in the snail host induced by concurrent heterospecific infection, toxic chemical compounds released by the larvae, or competition for nutrients or oxygen. This possibility appears to enhance super infection by only one parasite [31,32].

We depend on the identification to the family level and we gave these names for cercariae as they are new species.

## Conclusion

This study increases the importance of *L. littoria* snails as a source of infection of new parasites. Moreover, further epidemiological studies continued now to identify the adult worms of these three species of strigeid *Cercaria* to evaluate its effects on the probable final hosts if it is fish or water birds.

## Authors’ Contributions

FAB and MTA were in charge of designing the study and writing the manuscript. The samples were taken by MTA and MMA. The supervision of the laboratory work performed by FAB and MTA. All authors have read and approved the final manuscript.
